# The Upregulation of Caffeic Acid Phenethyl Ester on Growth Differentiation Factor 15 Inhibits Transforming Growth Factor β/Smad Signaling in Bladder Carcinoma Cells

**DOI:** 10.3390/biomedicines10071625

**Published:** 2022-07-07

**Authors:** Chen-Pang Hou, Ke-Hung Tsui, Syue-Ting Chen, Kang-Shuo Chang, Hsin-Ching Sung, Shu-Yuan Hsu, Yu-Hsiang Lin, Tsui-Hsia Feng, Horng-Heng Juang

**Affiliations:** 1 Graduate Institute of Clinical Medical Science, College of Medicine, Chang Gung University, Kwei-Shan, Taoyuan 33302, Taiwan; glucose1979@cgmh.org.tw; 2 Department of Urology, Chang Gung Memorial Hospital-Linkou, Kwei-Shan, Taoyuan 33302, Taiwan; laserep@mail.cgu.edu.tw; 3 Department of Healthcare Management, Yuanpei University of Medical Technology, Hsinchu 300, Taiwan; 4 Department of Urology, Shuang Ho Hospital, New Taipei City 235041, Taiwan; t2130@s.tmu.edu.tw; 5 TMU Research Center of Urology and Kindey, Department of Medicine, College of Medicine, Taipei Medical University, Taipei 11031, Taiwan; 6 Graduate Institute of Biomedical Sciences, College of Medicine, Chang Gung University, Kwei-Shan, Taoyuan 33302, Taiwan; stchen2021@mail.cgu.edu.tw (S.-T.C.); d000016684@cgu.edu.tw (K.-S.C.); hcs@mail.cgu.edu.tw (H.-C.S.); hsusy@mail.cgu.edu.tw (S.-Y.H.); 7 Department of Anatomy, College of Medicine, Chang Gung University, Kwei-Shan, Taoyuan 33302, Taiwan; 8 School of Nursing, College of Medicine, Chang Gung University, Kwei-Shan, Taoyuan 33302, Taiwan; thf@mail.cgu.edu.tw

**Keywords:** bladder, TGFβ, Smad, CAPE, GDF15, maspin, NDRG1

## Abstract

Growth differentiation factor 15 (GDF15) is known as a TGFβ-like cytokine acting on the TGFβ receptor to modulate target genes. GDF15 is regarded as a tumor suppressor gene in the human bladder and the caffeic acid phenethyl ester (CAPE) induces GDF15 expression to inhibit the tumor growth in vitro and in vivo. However, the interactions among GDF15, CAPE, and TGFβ/Smads signaling in the human bladder carcinoma cells remain unexplored. Results revealed that TGFβ downregulated the expression of GDF15 via the activation of Smad 2/3 and Smad 1/5. Induction of GDF15 on its downstream genes, NDRG1 and maspin, is dependent on the TGFβ/Smad pathways. Moreover, TGFβ blocked the CAPE-inducing expressions of GDF15, maspin, and NDRG1. Pretreatment of TGF receptor kinase inhibitor not only blocked the activation of TGFβ but also attenuated the activation of GDF15 on the expressions of maspin and NDRG1. The CAPE treatment attenuated the activation of TGFβ on cell proliferation and invasion. Our findings indicate that TGFβ downregulated the expressions of GDF15, maspin, and NDRG1 via TGFβ/Smad signaling. Whereas, CAPE acts as an antagonist on TGFβ/Smad signaling to block the effect of TGFβ on the GDF15 expression and cell proliferation and invasion in bladder carcinoma cells.

## 1. Introduction

Propolis, a natural resinous mixture produced by honey bees hives, has been well-known in possessing a broad spectrum of pro-healthy properties [1]. Caffeic acid phenethyl ester (CAPE), the major bioactive propolis compound, possesses most health-related features including anti-tumor [2]. The study has addressed that several biological signaling processes including Akt phosphorylation, mitogen-activated protein kinase (MAPK), and AMP-activated protein kinase (AMPK), NF-κB, and p53 pathways have involved in the antitumor effect of CAPE [3]; however, the modulation of CAPE in the expression of target genes occurs in different signals and is cell-dependent [4,5,6,7]. A recent study revealed that CAPE upregulated the expression of GDF 15 to inhibit the growth of bladder carcinoma cells [6].

Growth differentiation factor 15 (GDF15) is a secretory dimeric protein with a wide variety of tissue-specific and cell-specific presentations [8,9]. We know that GDF15 potentially represents a proper indicator of disease progression since the expression of GDF15 is associated with several health problems such as obesity, diabetes, cardiovascular disease, and cancers [10]. The effects of GDF15 in cancer cell metastasis function are divergent either in a highly tissue-specific manner or a cell-specific manner [11]. Prior studies have illustrated that GDF15 is upregulated by DNA demethylation and p53, and is regarded as a tumor suppressor gene in bladder cancer [12,13,14,15,16,17]. Moreover, our early report demonstrated that GDF15 is the upstream gene of two antitumor genes, NDRG1 and maspin, in bladder carcinoma cells [14].

Transforming growth factor (TGF) is a pleiotropic cytokine involved in cancer formation, fibrosis, and immunologic disorders [18]. The context-dependent signal of TGFβ signaling includes various positive and negative modifiers, receptors, and phosphorylation of Smad proteins (the downstream target of TGFβ receptor kinase) [19]. The expression and signaling of TGFβ/TGFβ receptors (TGFβRs) have long been recognized as a critical pharmacologic therapy for enhancing urinary bladder function [20,21]. However, the TGFβ signaling forms a complex web in the progress of different cancers, including in bladder cancer [22]. It is known that the function of TGFβ in normal and premalignant cells is tightly controlled to enforce homeostasis and suppress tumor progression. When cancer cells have lost the TGFβ tumor-suppressive response however, they can turn TGFβ to their advantage to establish and expand metastasis [23]. An early study suggested that TGFβ/TGFβRs signaling acts as a potent tumor suppressor in rat bladder carcinoma cells [24], however, other studies revealed that the TGFβ pathway is involved in the human bladder cancer progression [25,26,27,28]. Results of a xenograft animal study indicated that treatment of SB431542, an inhibitor of the TGFβ/Smad pathway, reduced the tumor growth of human bladder carcinoma cells in vivo [29]. These results highlighted the aspects of TGFβ signaling with relevance to the process of bladder cancer.

Our previous studies indicated that GDF15 is deemed as an antitumor gene in the human bladder carcinoma cells and CAPE treatment induces the expression of GDF15 via the MAPK and/or AMPK signaling [6,14]. As GDF15 possesses characteristic structures of cytokines in the TGFβ superfamily, studies have confirmed that GDF15 acts on the TGFβ receptor to modulate target genes [30,31]. A recent study revealed that GDF15 promotes the progression of esophageal squamous cells by increasing cell proliferation, migration, and invasion via TGFβRII signaling [32]. However, the interaction between GDF15 and TGFβ/Smads signaling in the human bladder is still unknown.

The objectives of this research are to determine the effect of TGF/Smads on the GDF15 expression and the role of CAPE as a TGF/Smads antagonist in human bladder cancer cells.

## 2. Materials and Methods

### 2.1. Cell lines and Cell Culture

The bladder carcinoma cell lines (RT-4, HT1376, and T24 cells) were obtained from the Bioresource Collection and Research Center (BCRC, Hsinchu, Taiwan) and cultured as described previously [6]. RPMI 1640 media was obtained from Invitrogen (Carlsbad, CA, USA). CAPE and SB431542 were purchased from Selleckchem (Houston, TX, USA). LY364947 was purchased from Sigma–Aldrich Co. (St. Louis, MO, USA). The CAPE was dissolved in the dimethyl sulfoxide (DMSO) at 60 mM as the stock solution. Recombinant human TGF-β1 (rhTGFβ) and recombinant human GDF15 (rhGDF15) were purchased from PEPRO TECH (Cranbury, NJ, USA) and R&D Systems Inc. (Minneapolis, MN, USA), respectively, and dissolved as the manufacture protocol.

### 2.2. Expression Vector Constructs and Stable Transfection

The expression vector of human GDF15 (pcDNA3-GDF15) was constructed and transfected into bladder carcinoma T24 cells by electroporation and selecting clonally with G418 as described previously [14].

### 2.3. Immunoblot Assay

Equal amounts of whole-cell lysates were separated on a 10–12% SDS-polyacrylamide gel. The blotting membranes were probed using antiserum of GDF15 (Ab206414, Abcam, Cambridge, MA, USA), NDRG1 (42-6200; Invitrogen, Carlsbad, CA, USA), Maspin (#554292, BD Biosciences, San Jose, CA, USA), GFRAL (ab107719, Abcam, Cambridge, MA, USA), β-actin (MAB1501, Merck Millipore, Burlington, MA, USA), Smad 2/3 (SC-398844, Santa Cruz Biotechnology, Santa Cruz, CA, USA), Smad 1 (#6944, Cell Signaling, Danvers, MS, USA), Smad 5 (#9517, Cell Signaling, Danvers, MS, USA), phospho-Smad 2/3 (#8828, Cell Signaling, Danvers, MS, USA), or phospho-Smad 1/5 (#9516, Cell Signaling, Danvers, MS, USA) antiserum. Band intensities were detected using the Western lightning plus-ECL detection system (PerkinElmer Inc., Waltham, MA, USA), recorded using the LuminoGraph II (Atto Corporation, Tokyo, Japan), and analyzed using the Image J [33].

### 2.4. Real-Time Reverse Transcriptase-Polymerase Chain Reaction

The total RNA was extracted from cells with Trizol reagent and cDNA was synthesized by using the superscript III preamplification system (Invitrogen). FAM dye-labeled TaqMan MGB probes as well as PCR primers, which are used for human GDF15 (Hs00171132_m1), NDRG1 (Hs00608387_m1), Maspin (Hs00985283_m1), GFRAL (Hs01087628_m1), and β-actin (Hs01060665_g1), and were purchased from Applied Biosystems. The real-time PCR (qPCR) was performed using a CFX Connect Real-Time PCR system (Bio-Rad Laboratories, Foster City, CA, USA) as described previously [6]. Mean cycle threshold (C_t_) values for target genes were normalized against the β-actin control probe to calculate Δ C_t_ values. All reactions were conducted on at least three independent occasions.

### 2.5. Enzyme Linked Immunosorbent Assay

Cells were incubated in a 0.5 mL of RPMI 1640 medium supplemented with 10% FCS for 24 h. The media were changed to serum-free RPMI 1640 with/without 10 ng/mL rhTGFβ, 20 μg/mL SB431542, or 20 μM CAPE as indicated. The GDF15 levels of the cell supernatant were measured by enzyme-linked immunosorbent assay kit (Catalog #: DY957; R&D Systems, Inc., Minneapolis, MN, USA) as described previously [14]. The protein levels in each sample were adjusted by the concentration of protein in the whole-cell extract, which was measured using a bicinchoninic acid protein assay kit (Pierce Protein Research, Rockford, IL, USA).

### 2.6. EdU Flow Cytometry Assay

Cells (1 × 10^5^) were cultured in RPMI 1640 medium with 10% FCS for 24 h, and then cultured in a serum-free medium for another 24 h. After being incubated with with/without 10 ng/mL TGFβ or 20 μM CAPE as indicated for another 24 h. The EdU (5-ethynyl-2′-deoxyuridine; 10 μM; Thermo Fisher Scientific Inc., Waltham, MA, USA) was added to the culture medium for 2 h. The EdU fluorescence of cells was counted using an Attune NxT acoustic focusing cytometer (Thermo Fisher Scientific Inc., Waltham, MA, USA).

### 2.7. WST-1 and CyQUANT Cell Proliferation Assay

Cell proliferation was measured by the *WST*-*1* assay kit (ab155902; Abcam, Cambridge, MA, USA) or CyQUANT cell proliferation assay kit (Invitrogen, Carlsbad, CA, USA). Briefly, three thousand cells were seeded into each well of a 96-well plate in RPMI 1640 medium with 10% FCS for 24 h. The culture media were changed to serum-free medium with/without 10 ng/mL TGFβ or 20 μM CAPE for 24 h. For WST-1 cell proliferation cells, a 10 μL of WST-1 reagent was added directly into the culture medium and cells were incubated for 1 h before the amount of formazan dye was produced by measuring the absorbance at 440 nm using the synergy H1 microplate reader (BioTek Instruments, Inc., Beijing, China). For the CyQUANT cell proliferation assay, after cells were washed twice with phosphate-buffered saline (PBS), the cell pellet was frozen at −80 °C for 1 h. Then, 200 μL of CyQUANT GR dye with lysis buffer (Invitrogen) was added to each well and incubated for 10 min at room temperature before the fluorescence was measured at 488 nm excitation using the synergy H1 microplate reader (BioTek Instruments Inc., Beijing, China).

### 2.8. Matrigel Invasion Assay

The cells were treated with/without 10 ng/mL rhTGFβ or 20 μM CAPE as indicated for 24 h. The invasion ability of cells was determined by the Matrigel invasion assay. The transmembrane was fixed with 4% paraformaldehyde, then stained with 0.1% crystal violet solution for 30 min. The numbers of cells that invaded the Matrigel were recorded microscopically and defined using the Image J program [33].

### 2.9. Reporter Vectors Construct, Transient Transfection, and Reporter Assay

The SBE4-Luc reporter vector (Addgene; plasmid #16495; Watertown, MA, USA) containing four copies of the Smad binding site was a gift from Dr. Yu-Sun Chang (Graduate Institute of Biomedical Sciences, Chang Gung University, Taiwan). The DNA fragment containing enhancer/promoter of the human GDF15 gene (−2887 to +3) was cloned as described previously [6]. Cells were seeded in a 24-well plate, and allowed to grow for 24 h prior to transfection. Cells were then transiently transfected with reporter vectors using the X-tremeGene HP DNA transfection reagent (Roche Diagnostics GmbH, Mannheim, Germany). After 24 h, the transfected cells were treated with/without 10 ng/mL rhTGFβ, 20 μM SB431542, or 20 μM CAPE as indicated in serum-free RPMI1640 media for another 24 h. The reaction was terminated by washing twice in PBS and then adding 200 μL of Luciferase Cell Culture Lysis Reagent (Promega Corporation, Madison, MI, USA). The luciferase activity was determined in a relative light unit (RLU) using a Synergy H1 microplate reader (BioTek Instruments Inc., Beijing, China) and adjusted by the concentration of protein in the whole-cell extract as described above.

### 2.10. Statistical Analysis

Results are expressed as the mean ± standard error (S.E.). Statistical significance was determined by Student *t*-test analysis and ANOVA. The multiple comparisons were conducted using ANOVA with Tukey’s post hoc test with the SigmaStat program for Windows version 2.03 (SPSS Inc., Chicago, IL, USA).

## 3. Results

### 3.1. Effect of TGFβ/Smad Signaling on the Expressions of GDF15, Maspin, and NDRG1 in Bladder Carcinoma Cells

**Figure 1:**
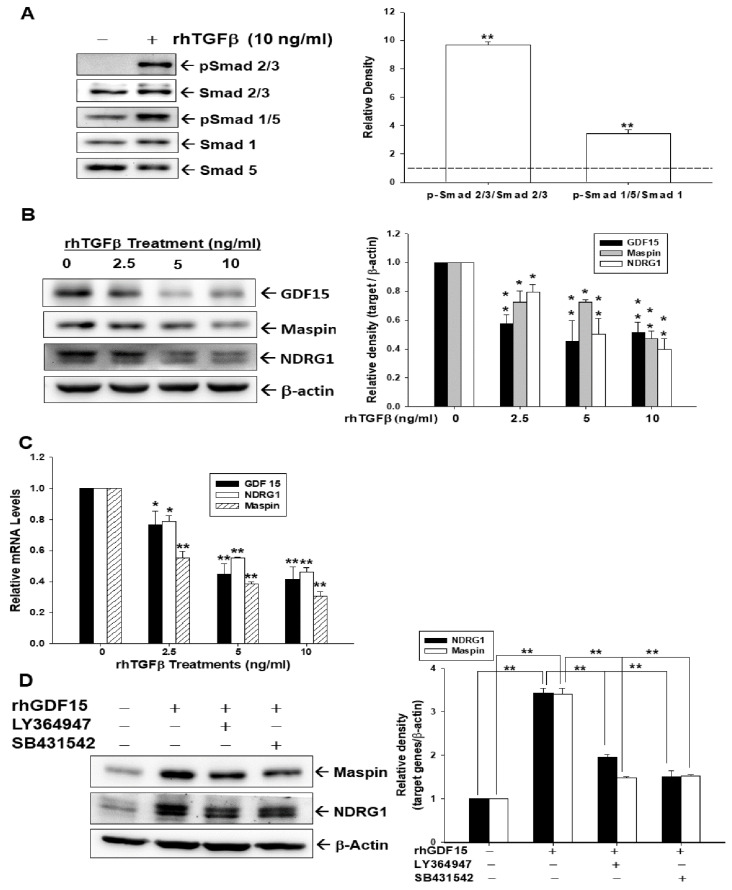
TGFβ downregulates expressions of GDF15, maspin and NDRG1 is dependent on TGFβ/Smad signaling in bladder carcinoma cells. The expressions of Smad 2/3, p-Smad 2/3, Smad 1, Smad 5, and p-Smad 1/5 were determined using immunoblot assays ((**A**), left) and quantitative analysis ((**A**), right) after treated with (+) or without (−) 10 ng/mL rhTGFβ for 1 h in HT1376 cells. The expressions of GDF15, maspin, NDRG1, and β-actin were determined using immunoblot assays ((**B**), left) and quantitative analysis ((**B**), right) after being treated with various concentrations of rhTGFβ as indicated for 18 h in HT1376 cells. (**C**) HT1376 cells were treated with various concentrations of rhTGFβ as indicated for 18 h. The relative mRNA levels of GDF15, NDRG1, and maspin were determined by RT-qPCR assays. (**D**) The expressions of maspin, NDRG1, and β-actin were determined using immunoblot assays ((**D**), left) and quantitative analysis ((**D**), right) after being treated with 400 ng/mL rhGDF15, 20 μM LY364947, and 20 μM SB431542 as indicated for 18 h. The quantitative data were expressed as the intensity of protein bands of the target genes/β-actin relative to the control solvent-treated group (*n* = 3). * *p* < 0.05, ** *p* < 0.01.

Results of the immunoblot assays showed that Smad 2/3 and Smad 1/5 were phosphorylated after 10 ng/mL of rhTGFβ treatment in HT1376 cells (Figure 1A, left). Results of quantitative analyses from three independent experiments indicated that rhTGFβ treatments induced 9.7-fold and 3.4-fold of Smad 2/3 and Smad 1/5 phosphorylation, respectively (Figure 1A, right). To explore whether the rhTGFβ treatments induced the expression of GDF15 and its downstream genes, maspin and NDRG1, we treated cells with 0–10 ng/mL of rhTGFβ in HT1376 cells. The treatment of rhTGFβ dosage-dependently decreased the protein levels of GDF15, maspin, and NDRG1, according to immunoblot assays and quantitative analyses (Figure 1B). RT-qPCR experiments yielded similar results (Figure 1C). To investigate whether rhGDF15 treatment induced expressions of maspin and NDRG1 in bladder carcinoma cells via the TGFβ/Smad pathways, HT1376 cells were pretreated for 1 h with SB431542 or LY364947, TGF receptor kinase inhibitors, before being exposed to rhGDF15. Interestingly, immunoblot assays and quantitative analyses further confirmed that both SB431542 and LY364947 pretreatments blocked the activation of rhGDF15 on the expressions of mapsin and NDRG1 (Figure 1D) indicating that the effect of GDF15 on maspin and NDRG1 expressions is dependent on the TGFβ/Smad pathways.

### 3.2. Effect of TGFβ on the Expressions of GDF15, Maspin, and NDRG1 Is Blocked by Pretreatment of SB431542 in Bladder Carcinoma Cells

**Figure 2:**
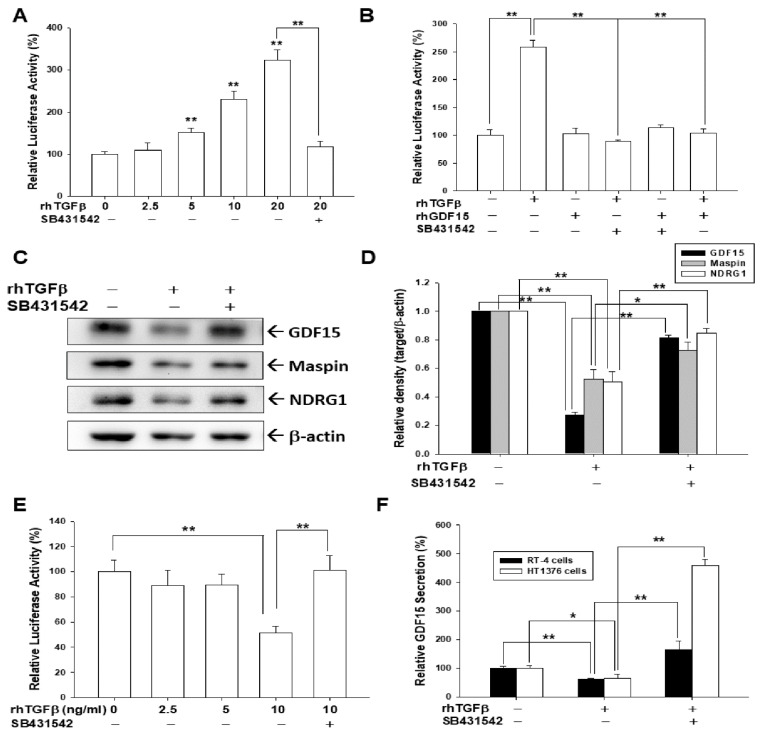
TGFβ receptor kinase inhibitor blocked the effect of TGFβ on the expressions of GDF15, NDRG1 and maspin in bladder carcinoma cells. The reporter activity of SEB4 reporter vector treated with (**A**) various concentrations of rhTGFβ or with 20 μM SB431542, or (**B**) 10 ng/mL rhTGFβ treatments with (+) or without (−) 400 nM rhGDF15 or 20 μM SB431542 pretreatment, as indicated, in the HT1376 cells. The expressions of GDF15, maspin, and NDRG1 were determined using immunoblot assays (**C**) and quantitative analysis (**D**) after being treated with (+) or without (−) 10 ng/mL rhTGFβ and 20 μM SB431542 for 18 h in HT1376 cells. The quantitative data were expressed as the intensity of protein bands of the target genes/β-actin relative to the control solvent-treated group (*n* = 3). (**E**) The reporter activity of the GDF15 reporter vector was treated with various concentrations of rhTGFβ or with 20 μM SB431542 as indicated. Data are expressed as the mean percentage of luciferase activity relative to the mock-treated group (*n* = 6). (**F**) RT-4 and HT1376 cells were treated with 10 ng/mL rhTGFβ with (+) or without (−) 20 μM SB431542 pretreatment for 24 h. The supernatants of culture media (*n* = 4) were collected and GDF15 secretions were determined by ELISA. The data were presented as the mean percentage compared with the control group. * *p* < 0.05, ** *p* < 0.01.

As rhTGFβ treatment upregulated Smad 1/5 and Smad 2/3 phosphorylation in HT1376 cells in Figure 1A, we continued to determine whether the effect of TGFβ on expressions of GDF15, Maspin, and NDRG1 was blocked by the SB431542, an inhibitor of TGFβ/Smad pathway. Reporter assays revealed that rhTGFβ treatments in HT1376 cells dosage-dependently upregulated the reporter activity of the SBE4-Luc reporter vector, which contains four copies of the Smad binding site; while pretreatment of SB431542 blocked the activation of rhTGFβ on reporter activity (Figure 2A). Further reporter assays showed that rhGDF15 treatment did not affect the reporter activity of the SBE4-Luc reporter vector; meanwhile, co-treatment of rhGDF15 blocked the activation of rhTGFβ on Smad reporter activity (Figure 2B). Immunoblot assays (Figure 2C) and quantitative analyses (Figure 2D) indicated that rhTGFβ treatment downregulated the amount of GDF15, maspin, and NDRG1, while pretreated with SB431542 attenuated the inhibitory TGFβ effect of those protein expressions. We continued to determine the effect of rhTGFβ on GDF15 expression. Reporter assays using the specific human GDF15 reporter vector revealed that rhTGFβ treatment downregulated the reporter activity of the GDF15 reporter vector; however, being pretreated with SB431542 reversed such an effect in bladder carcinoma HT1376 cells (Figure 2E). Similar results were found in the ELISA assays. The rhTGFβ treatment downregulated the GDF15 secretion and being pretreated with SB431542 reversed such an effect in bladder carcinoma HT1376 and RT-4 cells (Figure 2F). Taken together, our results indicated that TGFβ downregulated GDF15 expression via Smad pathways to block its downstream genes, maspin and NDRG1, in bladder carcinoma cells.

### 3.3. GDF15 Blocks the Effect of TGFβ/Smad Signaling on the Expressions of Maspin and NDRG1 in Bladder Carcinoma Cells

**Figure 3:**
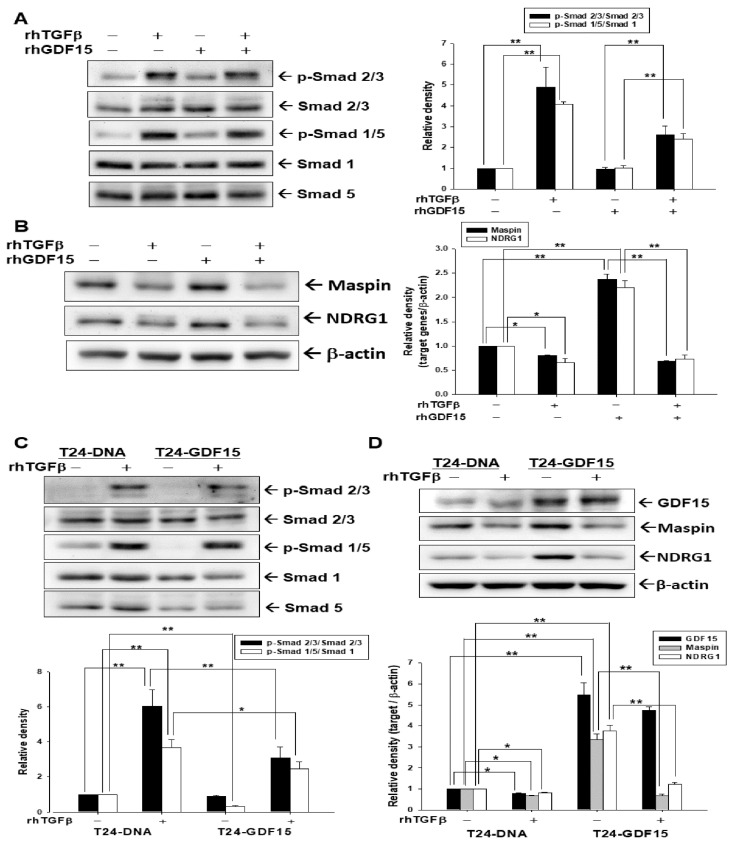
GDF15 inhibits the effect of TGFβ on Smas signaling activation and the expressions of GDF15, maspin, and NDRG1 in bladder carcinoma cells. The expressions of Smad 2/3, p-Smad 2/3, Smad 1, Smad 5, and p-Smad 1/5 were determined using immunoblot assays ((**A**), left) and quantitative analysis ((**A**), right) after treated with (+) or without (−) 10 ng/mL rhTGFβ and 400 ng/mL rhGDF15 as indicated for 1 h in T24 cells. The expressions of maspin, NDRG1, and β-actin were determined using immunoblot assays ((**B**), left) and quantitative analysis ((**B**), right) after being treated with (+) or without (−) 10 ng/mL rhTGFβ and 400 ng/mL rhGDF15 as indicated for 18 h in T24 cells. The expressions of Smad 2/3, p-Smad 2/3, Smad 1, Smad 5, and p-Smad 1/5 were determined using immunoblot assays ((**C**), top) and quantitative analysis ((**C**), bottom) after being treated with (+) or without (−) 10 ng/mL rhTGFβ as indicated for 1 h in T24-DNA and T24-GDF15 cells. The expressions of maspin, NDRG1, and β-actin were determined using immunoblot assays ((**D**), top) and quantitative analysis ((**D**), bottom) after treated with (+) or without (−) 10 ng/mL rhTGFβ and 400 ng/mL rhGDF15 as indicated for 18 h in T24-DNA and T24-GDF15 cells. The quantitative data were expressed as the intensity of protein bands of the p-target gene/target gene or target genes/β-actin relative to the control solvent-treated group (*n* = 3). * *p* < 0.05, ** *p* < 0.01.

We continued to explore whether the antagonistic effect between GDF15 and TGFβ is dependent on the Smad signaling pathway in bladder carcinoma cells. Immunoblot assays and quantitative analyses demonstrated that rhTGFβ increased Smad 2/3 and Smad 1/5 activations; however, the expressions of p-Smad 2/3 and p-Smad 1/5 decreased after co-treated with rhGDF15. The rhGDF15 alone did not affect the phosphorylation of Smad 2/3 and Smad 1/5 in bladder carcinoma T24 cells (Figure 3A). Further immunoblot assays and quantitative analyses revealed that maspin and NDRG1 protein levels were decreased when T24 cells were treated with rhTGFβ, while increased after rhGDF15 treatment (Figure 3B). Results of immunoblot and quantitative analyses showed that TGFβ induced the phosphorylation of Smad 2/3 and Smad 1/5 in mock-transfected T24 cells; however, GDF15 overexpression attenuated the TGFβ activation on phosphorylation of Smad 2/3 and Smad 1/5. Moreover, the endogenous Smad 1/5 activation in GDF15-overexpressed T24 cells was significantly lower than mock-transfected T24 cells (Figure 3C). The rhTGFβ treatment did not affect the amount of exogenous GDF15 in GDF15-overexpressed T24 cells. The rhTGFβ treatment indeed downregulated the maspin and NDRG1 expressions in mock-transfected and GDF15-overexpressed T24 cells, although exogenous GDF15 induced maspin and NDRG1 expressions in T24 cells (Figure 3D).

### 3.4. TGFβ Co-Treatment Blocks CAPE Inducing the Expressions of GDF15, Maspin, and NDRG1 in Bladder Carcinoma Cells

**Figure 4:**
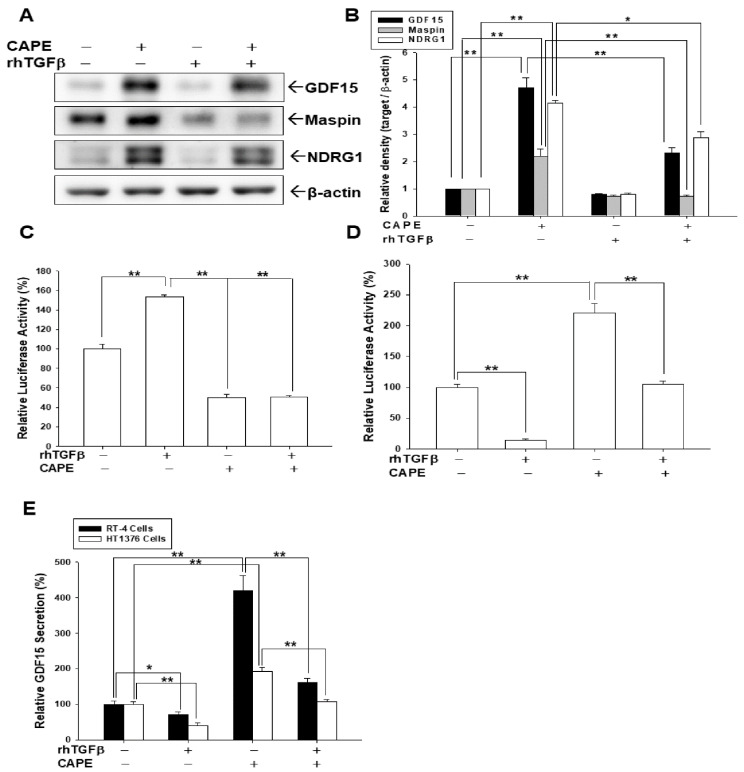
CAPE attenuates the decreasing effect of TGFβ on the expressions of GDF15, maspin, and NDRG1 in human bladder carcinoma cells. The expressions of GDF15, maspin, NDRG1, and β-actin were determined using immunoblot assays (**A**) and quantitative analysis (**B**) after being treated with (+) or without (−) 10 ng/mL rhTGFβ and 20 μM CAPE as indicated for 18 h in HT1376 cells. The quantitative data were expressed as the intensity of protein bands of the target genes/β-actin relative to the control solvent-treated group (*n* = 3). The reporter activity of SEB4 (**C**) and GDF15 (**D**) reporter vector treated with (+) or without (−) 10 ng/mL rhTGFβ and 20 μM CAPE as indicated in the HT1376 cells. Data are expressed as the mean percentage of luciferase activity relative to the control group (*n* = 6). (**E**) RT-4 and HT1376 cells were treated with 10 ng/mL rhTGFβ treatments with (+) or without (−) 20 μM CAPE pretreatment as indicated for 24 h. The supernatants of culture media (*n* = 4) were collected and GDF15 secretions were determined by ELISA. The data were presented as the mean percentage compared with the control group. * *p* < 0.05, ** *p* < 0.01.

To assess the antagonistic effect of CAPE on TGFβ treatments in bladder carcinoma cells, we treated HT1376 cells with CAPE and/or rhTGFβ to determine the expressions of GDF15, maspin, and NDRG1. Results of immunoblot assays (Figure 4A) and quantitative analyses (Figure 4B) showed that CAPE upregulated the expressions of GDF15, maspin, and NDRG1; however, TGFβ blocked the activation of CAPE on the protein levels of GDF15, maspin, and NDRG1. Similar results were found in the reporter assays. Results from reporter assays using the specific Smad reporter vector showed that rhTGFβ treatments upregulated the reporter activity; however, co-treatment with CAPE reversed such effect in bladder carcinoma HT1376 cells (Figure 4C). On the other hand, using the human GDF15 reporter vector, results of reporter assays revealed that rhTGFβ treatments downregulated the reporter activity, while co-treatment with CAPE reversed such effect in bladder carcinoma HT1376 cells (Figure 4D). CAPE stimulated the GDF15 secretion, while being co-treated with rhTGFβ blocked the activation of CAPE on GDF15 secretion from bladder carcinoma, RT-4 and HT1376, cells (Figure 4E).

### 3.5. CAPE Treatment Acts as the Antagonist of TGFβ/Smad Signaling in Bladder Carcinoma Cells

**Figure 5:**
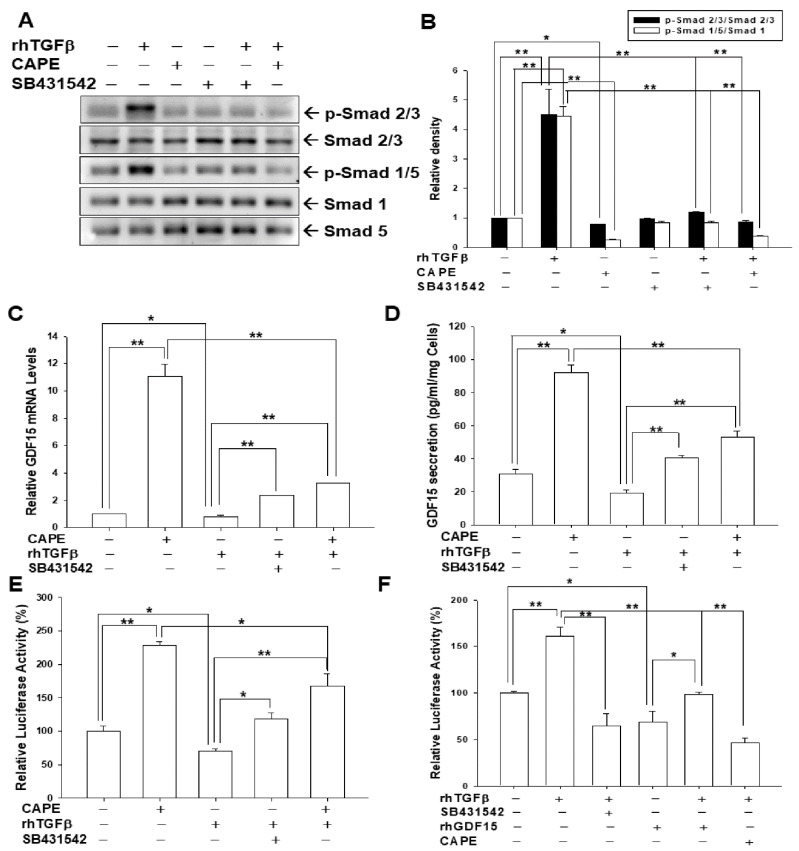
CAPE acts as the inhibitor of TGFβ receptor kinase in human bladder carcinoma cells. (**A**) The Smad 2/3, p-Smad 2/3, Smad 1, Smad 5, and p-Smad 1/5 expressions were determined using immunoblot assays after one hour of 10 ng/mL TGFβ treatments with (+) or without (−) 20 μM CAPE or 20 μM SB431542 pretreatment as indicated in T24 cells. (**B**) The quantitative data were expressed as the intensity bands of the phosphorylation proteins relative to the total protein levels for Smad 2/3 and Smad 1 (mean ± S.E.; *n* = 3). HT1376 cells were treated with 10 ng/mL rhTGFβ treatments with (+) or without (−) 20 μM CAPE or 20 μM SB431542 pretreatment as indicated for 18 h. (**C**) The relative mRNA levels (*n* = 3) of GDF15 were determined by RT-qPCR assays and (**D**) the supernatants of culture media (*n* = 4) were collected and GDF15 secretions were determined by ELISA. (**E**) The reporter activity of the GDF15 reporter vector treated with (+) or without (−) 10 ng/mL rhTGFβ, 20 μM CAPE or 20 μM SB431542, as indicated, in the HT1376 cells. (**F**) The reporter activity of the SEB4 reporter vector treated with 10 ng/mL rhTGFβ treatments with (+) or without (−) 20 μM CAPE or, 20 μM SB431542, or rhGDF15 pretreatment as indicated, in the HT1376 cells. Data are expressed as the mean percentage of luciferase activity relative to the mock-treated group (*n* = 6). * *p* < 0.05, ** *p* < 0.01.

To assess the CAPE treatments involved in Smad pathways in bladder carcinoma cells, we determined whether CAPE treatment attenuated the TGFβ-induced phosphorylation of Smad 2/3 and Smad 1/5. Results of immunoblot assays (Figure 5A) and quantitative analyses (Figure 5B) revealed that CAPE, which acts similarly to SB431542, downregulated the induction of rhTGFβ on the phosphorylation of Smad 2/3 and Smad 1/5. Results of RT-qPCR indicated that CAPE not only upregulated GDF15 gene expression but also blocked the decreasing effect of rhTGFβ on GDF15 gene expression (Figure 5C). Similar results were found in the ELISA assays (Figure 5D) and reporter assays using the human GDF15 reporter vector (Figure 5E). Further reporter assays using the Smad specific reporter vector confirmed that rhGDF15, CAPE, or SB431542 treatments blocked the activation of rhTGFβ on the Smad reporter activity (Figure 5F).

### 3.6. The Activation of TGFβ on Cell Proliferation and Invasion Is Attenuated by CAPE Treatment in Bladder Carcinoma T24 Cells

**Figure 6:**
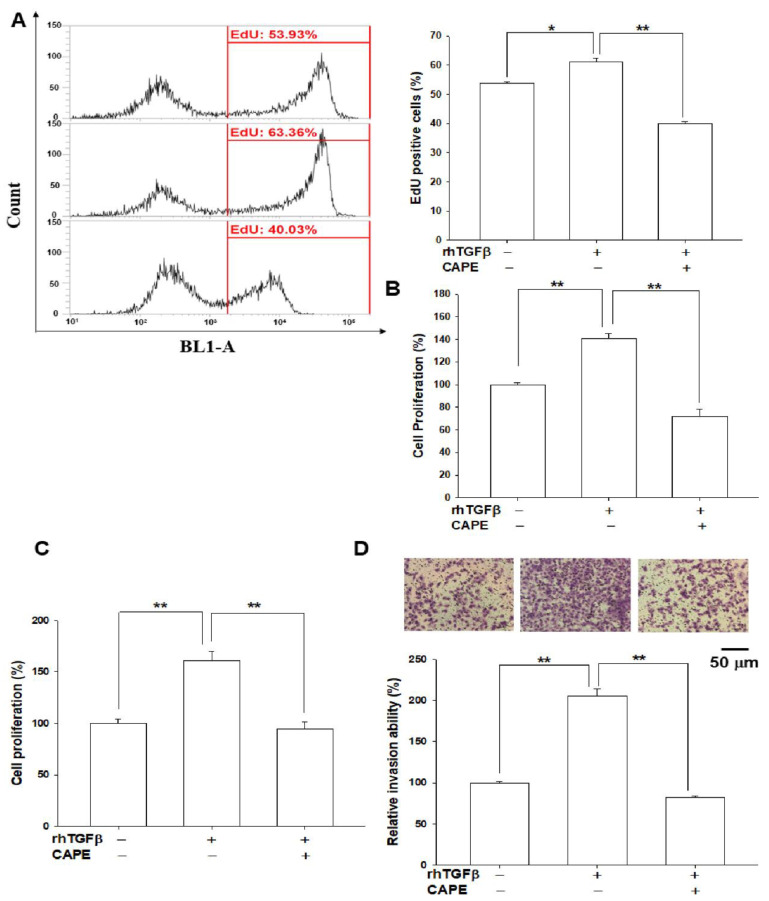
CAPE blocks the enhancement of TGFβ on cell proliferation and invasion. Cell proliferation of T24 cells after treated with rhTGFβ (10 ng/mL) and/or CAPE (20 μM) as indicated for 48 h was determined by EdU ((**A**), *n* = 4), WST-1 ((**B**), *n* = 8), and CyQUANT ((**C**), *n* = 8) assays. The data were presented as the mean percentage (±S.E.) compared with the control group. (**D**) T24 cells were treated with rhTGFβ (10 ng/mL) or rhTGFβ with CAPE (20 μM) for 24 h and the cell invasive ability was measured by the matrigel invasion assay after 24 h of incubation. The data were presented as the mean percentage (±S.E.) compared with the control group (*n* = 3). * *p* < 0.05, ** *p* < 0.01.

The results of cell proliferation determined by the 5′-ethynyl-2′deoxyuridine (EdU) flow cytometry assays revealed that the percentage of positive cells with EdU staining was increased by 8% in the TGFβ-treated T24 cells, while co-treatments of TGFβ and CAPE had a 22% decrease in EdU-positive cells compared to the vehicle group (Figure 6A). Similar results were found in WST-1 (Figure 6B) and CyQUANT (Figure 6C) cell proliferation assays. Results showed that treatment of TGFβ increased 40–61% cell proliferation compared to the vehicle-treated T24 cells, while co-treatment of CAPE attenuated 67–70% of the TGFβ treatment. Matrigel invasion assays revealed that TGFβ treatments induced about two-fold increase in cell invasive ability in T24 cells. However, the invasion capacity was downregulated by 123% in co-treatments of TGFβ and CAPE, when compared with TGFβ-treated T24 cells (Figure 6D).

## 4. Discussion

Growth differential factor 15 (GDF15), known as a TGF-like cytokine, is one of the factors that regulate cell development and differentiation [34]. The GDF15 holds a various range of tissue-specific and cell-specific presentations in cancerous biology [10,35]. Our previous studies have verified that both human bladder epithelial and stromal cells secret GDF15 in vitro [6]. Meanwhile, the results illustrated that GDF15 modulates epithelial-mesenchymal transition (EMT) markers NDRG1, and maspin to suppress the cellular proliferation, invasion, and growth of bladder carcinoma cells in vitro and in vivo, and is defined as an antitumor marker of bladder cancer [6,14].

Prior cellular metabolic studies recognized glial cell-derived neurotrophic factor family receptor-like (GFRAL), an orphan receptor in the GDNF family, as the GDF 15 receptor [36,37,38,39]; thereafter, evidences suggesting GDF15/GFRAL/RET (ret proto-oncogene signaling) is a target for treatment of metabolic diseases. Nevertheless, the expression of GFRAL and GDF15/GFRAL downstream signaling in cancers are yet to be discovered. To date, GFRAL expression is only identified in certain cells, tissues, and organs [37] and none of the reports showed the expression of GFRAL in human bladder cells in vitro or in vivo. Reports of RT-qPCR and immunoblot assays of bladder carcinoma cells (RT-4, HT1376, and T24) failed to identify the expression of GFRAL in these cells (data not shown).

A solid evidence has indicated that there is downregulation of transforming growth factor β (TGFβ)-signaling in tumor initiation, while upregulation of TGFβ-signaling has the ability to promote tumor progression in certain tumors [23,40]. Therefore, TGFβ is a highly pleiotropic cytokine and has a complex role in tumorigenesis. The TGFβ signaling is dependent on the receptors and the Smad proteins [19,23,41]. A recent discovery of cell contextual determinants suggested that some transcriptional modulators of Smads are critical to switching TGFβ responses from pro-apoptosis to pro-metastasis [42]. Early reports concerning the function of TGFβ in the bladder were controversial [24,25,26]. However, recent in vitro and in vivo studies suggested that TGFβ signaling is relevant to the process of bladder cancer [28,29,43,44,45]. In the present study, we found that TGFβ induced Smad 2/3 and Smad 1/5 phosphorylation in the human bladder carcinoma cells (Figure 1, Figure 3 and Figure 5), which is in line with the findings in early reports on epithelial and stromal cells of several different organs, including the bladder [41,46,47,48,49,50]. Our study revealed that TGFβ promoted bladder cancer cellular proliferation and invasion in vitro (Figure 6), which is consistent with the findings of other research regarding bladder urothelial carcinoma [51,52,53].

Reports have highlighted that GDF15 possesses characteristic structures of TGFβ superfamily protein and acts on the TGFβ receptor via TGFβ/Smad signaling or an alternative pathway to modulate target genes [32,34,35,54]. In the present study, our results showed that GDF15 and its downstream genes, maspin and NDRG1 were downregulated by TGFβ through the Smad 2/3 and Smad 1/5 activation in bladder carcinoma cells (Figure 1). Interestingly, our study is the first report which revealed that GDF15 blocked the effect of TGFβ/Smad signaling on the maspin and NDRG1 expressions in bladder carcinoma cells although GDF15 did not affect the phosphorylation of Smad 2/3 and Smad 1/5 in bladder carcinoma cells (Figure 3). Further study confirmed that TGFβ/Smad signal involved in the upregulation of GDF15 on the expressions of maspin and NDRG1 in bladder carcinoma cells, since being pretreated with the inhibitor of TGFβ/Smad pathway, SB431542 or LY364947, which not only blocked the activation of TGFβ on the phosphorylation of Smad 2/3 and Smad 1/5 but also attenuated both the inhibitory effect of TGFβ and activation of GDF15 on the expressions of maspin and NDRG1 in bladder carcinoma cells (Figure 1, Figure 2 and Figure 5). These results are similar to other studies, which showed the treatment of SB431542 decreased the tumor growth of human bladder in vitro and in vivo [29,54]. Caffeic acid phenethyl ester (CAPE) is a primary pharmacological-active component of propolis [1,2]. Our recent study confirmed the anti-tumor effect of CAPE on bladder cancer in vivo using the xenograft animal study [6]. Several possible molecular signaling pathways for the therapeutic application of CAPE in cancers including NFκB, p53, Wnt/β-catenin, PI3K/Akt, MAPK (mitogen-activated protein kinase), AMPK (AMP-activated kinase), and so forth have been discussed [3,6,7]. A study reported that caffeic acid phenylethyl amide decreased TGFβ-induced phosphorylation of Smad 2/3 in renal tubular epithelial NRK52E cells [55]. Another in vivo study indicated that CAPE-treatment decreased TGFβ^+^ and pSmad2^+^ cells in the breast tumor tissue of the irradiated mice [56]. However, the direct effect of CAPE on the activation of TGFβ has not yet been investigated. Our recent study revealed that CAPE treatment inhibits proliferation, invasion, and tumor formation of bladder carcinoma cells in vitro and in vivo by upregulating GDF15; moreover, the CAPE–induced treatment on the mapsin and NDRG1 expressions is GDF15 dependent, which is activated via the MAPK or AMPK signaling pathway [6]. Therefore, we continued to investigate whether TGFβ or CAPE modulated GDF15 and its target genes through the TGFβ/Smad signaling in the human bladder carcinoma cells. Our findings of immunoblot and reporter assays confirmed that downregulation of TGFβ on the GDF15, NDRG1, and maspin expressions is dependent on the Smads singling (Figure 2) and CAPE, acting similarly with SB431542, blocking the activation of TGFβ on Smad 2/3 and Smad 1/5 phosphorylation; furthermore, both CAPE and SB431542 blocked the decreasing effect of TGFβ on GDF15 expression and secretion (Figure 4 and Figure 5). CAPE treatment also inhibited the enhancement of TGFβ on cell proliferation and invasion (Figure 6). Taken together, these results suggested that CAPE acts as the inhibitor of TGFβ receptor kinase to block the activation of TGFβ on cell proliferation and invasion in bladder carcinoma cells. The present study is the first study to indicate that GDF15 and CAPE are the agents that possibly act against the TGFβ/Smad signaling in the human bladder carcinoma cells.

## 5. Conclusions

TGFβ/Smad signaling has a role in bladder cancer progression, and GDF15 is an antitumor gene in the human bladder. CAPE induces GDF15 expressions against the tumor growth of the human bladder carcinoma cells. Results of present studies implicate that TGFβ downregulates the expression of GDF15 and its downstream genes, maspin and NDRG1, via the activation of Smad 2/3 and Smad 1/5. CAPE acts as the inhibitor of TGFβ receptor kinase to block the enhancement of TGFβ on cell proliferation and invasion. Collectively, our study suggests that GDF15 and CAPE are the potential antagonists for the TGFβ/Smad signaling in the human bladder carcinoma cells.

## Data Availability

The data used to support the findings of this study are available from the corresponding author upon request.
